# Malondialdehyde and Oxidative Stress in Cancer: Biological Insights and Clinical Perspectives

**DOI:** 10.3390/biomedicines14081696

**Published:** 2026-07-28

**Authors:** Federica Li Pomi, Maria Clara Gama de Souza Silva, Giuseppe Murdaca, Francesco Borgia, Adele Bottaro, Sebastiano Gangemi, Alessandro Allegra

**Affiliations:** 1 Department of Precision Medicine in Medical, Surgical and Critical Care (Me.Pre.C.C.), University of Palermo, 90127 Palermo, Italy; federicalipomi@hotmail.it; 2 Allergy and Clinical Immunology Unit, Department of Clinical and Experimental Medicine, University of Messina, 98125 Messina, Italy; claragamasouza@gmail.com (M.C.G.d.S.S.); sebastiano.gangemi@unime.it (S.G.); 3 Department of Internal Medicine, University of Genova, 16132 Genova, Italy; 4 Allergology and Clinical Immunology Unit, San Bartolomeo Hospital, 19038 Sarzana, Italy; 5 Section of Dermatology, Department of Clinical and Experimental Medicine, University of Messina, 98125 Messina, Italy; fborgia@unime.it; 6 Division of Hematology, Department of Human Pathology in Adulthood and Childhood “Gaetano Barresi”, University of Messina, 98125 Messina, Italy; bottarp15@gmail.com (A.B.); alessandro.allegra@unime.it (A.A.)

**Keywords:** malondialdehyde, lipid peroxidation, oxidative stress, cancer, skin cancer, colorectal cancer, lung cancer, cervical cancer

## Abstract

Malondialdehyde (MDA) is one of the main end-products of lipid peroxidation (LPO) and represents a widely investigated marker of oxidative stress (OS) and oxidative tissue damage. Beyond its role as a measurable byproduct of polyunsaturated fatty acid peroxidation, MDA can interact with proteins and nucleic acids, generating adducts that may contribute to mutagenic, genotoxic, and cytotoxic events involved in carcinogenesis and tumor progression. This narrative review summarizes current evidence on the role of MDA in cancers, including breast, lung, head and neck, colorectal, cervical, and cutaneous tumors. Across these cancer types, increased circulating or tissue MDA levels have frequently been associated with enhanced LPO, impaired antioxidant defenses, tumor burden, advanced disease stage, aggressive histopathological features, treatment-related oxidative injury, and, in selected studies, poorer clinical outcomes. MDA-derived DNA adducts may further reflect oxidative DNA damage and provide mechanistic insight into the relationship between chronic redox imbalance, inflammation, and malignant transformation. However, MDA remains a non-specific biomarker influenced by age, smoking, diet, metabolic disorders, systemic inflammation, comorbidities, treatment exposure, and analytical methodology. Current evidence therefore supports MDA as a biologically relevant indicator of oxidative damage rather than a validated stand-alone diagnostic, prognostic, or therapeutic biomarker. Larger prospective studies using standardized and specific analytical methods are needed to clarify its clinical utility and to integrate MDA within broader redox biomarker panels.

## 1. Introduction

Reactive oxygen species (ROS) play a dual and context-dependent role in cancer biology. At controlled concentrations, they act as physiological signaling mediators, whereas persistent ROS accumulation may promote oxidative molecular damage, oncogenic signaling, and tumor progression. Conversely, when ROS levels exceed the antioxidant capacity of cancer cells, they can induce irreversible cellular injury and death [1].

### 1.1. Oxidative Stress

Oxidative stress (OS) is generally defined as an imbalance between the generation of ROS and cellular antioxidant defenses [2]. Under physiological conditions, low ROS levels also serve essential signaling functions, including the regulation of cell growth, migration, and differentiation [3].

Living organisms have finely tuned systems that regulate ROS levels, ensuring that production and removal remain balanced, thereby maintaining a steady-state concentration of ROS [4]. Redox homeostasis may be disrupted by increased ROS generation, depletion or inactivation of antioxidant systems, or the simultaneous activation of multiple pro-oxidant mechanisms. When antioxidant systems adequately counteract a transient increase in ROS, redox homeostasis may be restored [5,6,7]. However, if cells fail to neutralize excess ROS or return to their baseline redox state, ROS levels may remain persistently elevated, or the homeostatic set point may shift [8,9]. This sustained pro-oxidant state promotes oxidative modifications of cellular components and disrupts homeostasis, a condition referred to as chronic oxidative stress [10].

Persistent OS is involved in several pathological conditions [11,12,13,14,15,16,17,18]. In cancer, chronic redox imbalance may affect both malignant cells and the surrounding microenvironment, contributing to genomic instability, altered cellular signaling, inflammation, and tumor progression [1].

The balance between ROS production and elimination is maintained by a complex, multi-layered antioxidant system that removes ROS or limits their harmful effects. Antioxidant defenses include both low-molecular-weight compounds and high-molecular-weight enzymatic systems [19]. Low-molecular-weight antioxidants (non-enzymatic) are small molecules that directly scavenge free radicals or prevent their formation, including vitamin C, vitamin E, glutathione (GSH), carotenoids, uric acid, bilirubin, flavonoids, and polyphenols. High-molecular-weight (enzymatic) antioxidant systems include enzymes that catalytically eliminate ROS and constitute the primary endogenous antioxidant defense, including superoxide dismutase (SOD), catalase (CAT), glutathione peroxidase (GPx), glutathione reductase (GR), and thioredoxin reductase systems [20,21].

Among these systems, GSH is a major endogenous antioxidant that regulates cellular redox status either by directly interacting with reactive species or by acting as a co-substrate for ROS-detoxifying enzymes [22]. GSH-dependent enzymes are also involved in the reduction in lipid hydroperoxides, thereby linking antioxidant homeostasis to the control of lipid peroxidation (LPO) [23].

ROS originate from both internal and external sources. Endogenous sources include inflammatory cells, mitochondria, and peroxisomes [24]. Ionizing radiation and several exogenous or iatrogenic factors, including environmental pollutants, chemical agents, alcohol, dietary factors, tobacco smoke, chemotherapeutic agents, and infectious agents, may also contribute to ROS production. Several of these exposures are also implicated in carcinogenesis through DNA damage, inflammation, and sustained redox imbalance [8]. Therefore, the effects of ROS in cancer reflect the interaction among their source, intensity, duration, cellular localization, and tumor-specific antioxidant adaptations.

### 1.2. Lipid Peroxidation and MDA Formation: Mechanisms and Biological Consequences

Among ROS-mediated processes, LPO represents a major mechanism of oxidative damage [25].

Lipid peroxides exert cytotoxic effects through two main mechanisms: they disrupt the composition, structure, and dynamics of cellular membranes, thereby impairing membrane integrity; and they promote additional ROS formation or decompose into secondary reactive compounds capable of crosslinking DNA and proteins, leading to cellular damage [26,27,28].

LPO is a highly damaging process that preferentially oxidizes polyunsaturated fatty acids (PUFAs), such as linoleic, arachidonic, and docosahexaenoic acids, which contain bis-allylic methylene groups that are particularly susceptible to hydrogen abstraction [29]. This oxidation occurs through two main cellular pathways: a tightly regulated enzymatic pathway involving lipoxygenases, cyclooxygenases, and cytochromes P450, and an uncontrolled, non-enzymatic radical chain reaction. Intracellular labile iron pools can facilitate non-enzymatic initiation of LPO through Fenton chemistry, in which ferrous iron reacts with hydrogen peroxide to generate highly reactive hydroxyl radicals [29,30].

In both pathways, hydrogen abstraction from a bis-allylic carbon generates a carbon-centered lipid radical, which rapidly reacts with molecular oxygen to form a lipid peroxyl radical. The newly formed peroxyl radicals continuously abstract hydrogen atoms from adjacent membrane lipids, generating a cycle that produces primary lipid hydroperoxides and promotes extensive membrane damage. This cascade continues until radical–radical annihilation occurs or until it is terminated by endogenous chain-breaking antioxidants [31].

Because primary lipid hydroperoxides are relatively unstable, they can undergo transition-metal-catalyzed cleavage and β-fragmentation, generating cytotoxic secondary carbonyl compounds, including MDA, particularly from highly unsaturated fatty acids such as arachidonic acid [32]. Alternatively, they may be reduced to the corresponding lipid alcohols or decompose into reactive aldehydes [33]. GPx enzymes, especially GPX4, regulate LPO by using GSH to reduce phospholipid hydroperoxides to the corresponding phospholipid alcohols [34].

Aldehydes represent a major class of LPO degradation products, with 4-hydroxynonenal (4-HNE) and malondialdehyde (MDA) being the best-described and most extensively studied members. For this reason, LPO degradation products are widely used as markers for detecting and quantifying lipid oxidative damage in biological samples [35,36].

Peroxidation of membrane lipids substantially alters the physical properties of lipid bilayers. It modifies lipid–lipid interactions, membrane permeability, ion gradients, and membrane fluidity [37]. Peroxidized phospholipids may also undergo structural reorientation within the membrane, reducing membrane thickness and fluidity and modifying their interactions with surrounding cells [38,39,40].

MDA is a dialdehyde that reacts with primary amines in proteins and DNA, forming adducts and crosslinks. It can also generate 1,4-dihydropyridine adducts with primary amines [41]. In the oncological setting, MDA-derived protein and DNA adducts may provide a potential mechanistic link between persistent LPO, genomic damage, altered protein function, and malignant transformation.

Because MDA is relatively stable, it may diffuse from its site of formation and interact with proteins and nucleic acids at distant cellular sites. MDA may therefore represent both a marker of oxidative damage and a potential mediator linking persistent LPO to genomic injury, altered protein function, tumor progression, and treatment-related tissue damage [35].

Despite the extensive use of MDA as an OS biomarker, its biological and clinical interpretation in cancer remains uncertain. Available studies differ substantially in biological matrix, sampling procedures, analytical methods, tumor stage, treatment status, and control-group selection, limiting direct comparisons and preventing definitive conclusions regarding its diagnostic, prognostic, or predictive value. Recent advances in redox biology, ROS-modulating therapies, biomarker standardization, and precision oncology provide a timely rationale for reassessing whether MDA can progress from a nonspecific indicator of oxidative damage to a clinically informative cancer biomarker [42].

Building on previous literature, this review aims to provide a comparative overview of MDA-related evidence across breast, lung, head and neck, colorectal, cervical, and cutaneous malignancies. It integrates mechanistic data on MDA-derived adducts with clinical findings from circulating and tissue measurements, while considering biological confounders, analytical heterogeneity, and the distinction between association and clinical validation. By summarizing consistent and discordant findings, it aims to clarify the current evidence and identify areas for future standardized studies.

## 2. Search Strategy

A narrative literature search was performed in PubMed up to 30 April 2026 using combinations of MeSH terms and free-text keywords related to MDA, OS, and the selected malignancies. Original English-language studies published from 1980 onward were considered for the cancer-specific clinical synthesis. Reviews and systematic reviews were used to identify additional relevant literature and to support the general mechanistic and methodological background. Articles were selected according to their relevance to MDA formation, measurement, biological effects, or clinical associations in cancer.

## 3. ROS and LPO in Oncological Diseases

OS and chronic inflammation are closely interdependent processes involved in cancer development and progression. Excessive ROS and free radical production can significantly disrupt the structure and function of essential cellular components—including proteins, structural lipids, and nucleic acids—leading to extensive tissue damage. As a result, the chemical byproducts of this oxidative injury can serve as useful indicators to assess systemic and localized oxidative stress across different human cancers [25,27]. Among these byproducts, MDA is particularly relevant because it is a major product of LPO, can form biologically active adducts, and can be measured in circulating and tissue samples [43].

ROS participate in the different stages of carcinogenesis. During tumor initiation, ROS may generate oxidative DNA lesions, strand breaks, and mutagenic adducts, thereby promoting genomic instability. During tumor promotion, redox-sensitive signaling pathways sustain cellular proliferation, chronic inflammation, resistance to apoptosis, and clonal expansion [44,45]. During tumor progression, ROS may contribute to metabolic adaptation, angiogenesis, extracellular matrix remodeling, invasion, and metastatic dissemination [44,45,46]. Conversely, excessive ROS accumulation may exceed the antioxidant capacity of cancer cells, inducing apoptosis, ferroptosis, or other forms of cell death, thereby creating a potential therapeutic vulnerability [47]. These processes may influence the extent of LPO and, consequently, MDA generation in the tumor and its microenvironment.

During malignant transformation, neoplastic cells exhibit distinct redox and metabolic phenotypes, characterized by increased intracellular ROS production and altered antioxidant defenses. Both intrinsic metabolic changes and external environmental influences drive persistent ROS generation in tumor cells, thereby contributing to gene mutations, transcriptional dysregulation, altered cellular signaling, tumor development, and disease progression [44,45,48]. At the same time, cancer cells may increase antioxidant and detoxification systems to maintain ROS at levels that support proliferation and adaptation without reaching a lethal threshold. This dynamic balance contributes to tumor survival but may also generate dependence on specific redox-regulatory mechanisms [44,45].

Cellular and biochemical interactions within the complex tumor microenvironment strongly contribute to redox imbalance, specifically through bidirectional crosstalk among cancer cells, cancer-associated fibroblasts (CAFs), tumor-associated macrophages (TAMs), and intratumoral hypoxia [49]. For example, TAMs can generate ROS through NADPH oxidase activity. This macrophage-driven oxidative stress may contribute to the stabilization of hypoxia-inducible factor 1-alpha (HIF-1α) and to increased expression of downstream mediators, such as vascular endothelial growth factor (VEGF), thereby promoting angiogenesis and tumor growth. At the same time, cancer-associated fibroblasts (CAFs) in the stroma contribute to the ROS load, facilitating tumor cell migration, remodeling of the extracellular matrix, and metastasis through multiple mechanisms, including the secretion of matrix metalloproteinases (MMPs) and inflammatory cytokines. Moreover, under intratumoral hypoxia, mitochondrial complex III-derived ROS can contribute to HIF-1α stabilization, whereas NADPH oxidase-derived ROS from tumor and stromal cells may further reinforce redox-sensitive signaling. HIF-1α activation subsequently promotes VEGF expression, metabolic adaptation, angiogenesis, and, in selected tumor contexts, invasive behavior [50,51,52].

**Figure 1:**
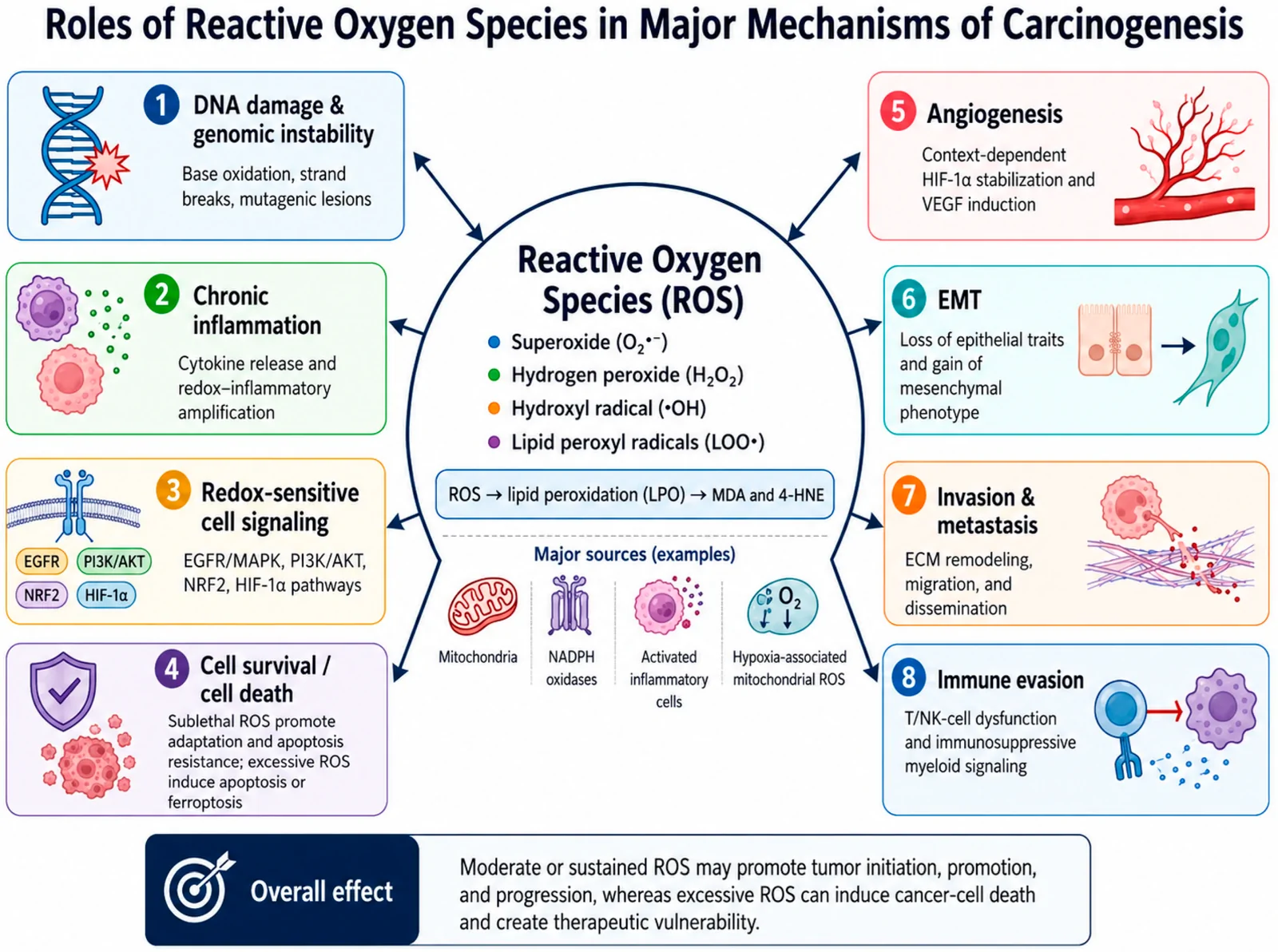
Role of ROS in major mechanisms of carcinogenesis.

Altered redox homeostasis and context-dependent ROS production can modulate several cancer-relevant signaling networks, including the epidermal growth factor receptor (EGFR)–RAS–rapidly accelerated fibrosarcoma (RAF)–mitogen-activated protein kinase (MEK)–extracellular signal-regulated kinase (ERK) pathway; the phosphatidylinositol 3-kinase (PI3K)–protein kinase B (AKT)–mechanistic target of rapamycin (mTOR) pathway; the Kelch-like ECH-associated protein 1 (KEAP1)–nuclear factor erythroid 2-related factor 2 (NRF2) axis; the HIF-1α–VEGF axis; and the ataxia-telangiectasia mutated (ATM)-dependent DNA-damage and oxidative-stress responses, including p53 signaling [53]. In growth factor-dependent pathways, ROS can transiently amplify signaling through direct and reversible oxidative modification of selected receptor tyrosine kinases, such as EGFR, and through reversible inhibition of redox-sensitive protein tyrosine phosphatases. ROS may also influence HIF-1α stabilization and ATM activation, although these effects depend on the cellular context, ROS source, and oxygen availability [54]. In established tumors, sustained or constitutive NRF2 activation can promote antioxidant and metabolic adaptation, tumor-cell survival, and treatment resistance by maintaining ROS at sublethal, signaling-compatible levels and limiting oxidative damage [55,56,57,58,59]. Depending on pathway status and cellular context, these redox-regulated responses may promote proliferation, metabolic adaptation, angiogenesis, and survival, or, conversely, induce cell-cycle arrest, senescence, and cell death [60,61] (Table 1, Figure 1).

The dual role of ROS also has therapeutic implications. Radiotherapy and several chemotherapeutic agents partly exert their anticancer activity by increasing oxidative damage. In parallel, inhibition of antioxidant defenses or lipid peroxide-detoxifying systems may raise ROS above the tolerance threshold of cancer cells. However, excessive systemic redox modulation may also damage non-malignant tissues, indicating that the therapeutic outcome depends on selectively exploiting the greater oxidative burden and redox dependency of tumor cells [62].

Within this redox framework, LPO represents a particularly relevant link between oxidative injury and MDA formation. A reciprocal relationship exists between OS, specifically LPO, and localized inflammation in oncological conditions [27]. Tumor tissue often undergoes lipid metabolic reprogramming, a characteristic of malignancy; to survive hypoxic, nutrient-poor environments, cancer cells increase the expression of enzymes involved in de novo fatty acid synthesis, mainly via sterol regulatory element-binding proteins (SREBPs). This metabolic reprogramming may alter membrane lipid composition and, consequently, the susceptibility of tumor cells to LPO and MDA generation. By modifying functional proteins, including metabolic enzymes and surface receptors, LPO-derived adducts can destabilize protein structures and interfere with molecular interactions. These modifications allow reactive lipid species to trigger distinct pathological responses in tumor cells and infiltrating immune cells [63,64]. Changes in the synthesis and composition of membrane lipids may also affect the susceptibility of tumor cells to oxidative injury and lipid peroxide accumulation.

For example, 4-HNE, a highly reactive and extensively studied LPO product, can influence tumor cell growth and differentiation processes [65]. At the plasma membrane interface, 4-HNE can inactivate membrane-associated catalase in tumor cells, thereby amplifying localized LPO and triggering hydroxyl radical-mediated apoptosis [66,67].

MDA, a major end-product of PUFA peroxidation, serves as a relevant biochemical marker in this landscape. Unlike short-lived free radicals, MDA is relatively stable, allowing it to diffuse within or out of the cell and interact with biological targets distant from the original site of free-radical injury [35,36]. Furthermore, MDA is not only a marker of LPO; it also forms as a metabolic byproduct of cyclooxygenase activity in platelets. This additional source should be considered when interpreting circulating MDA concentrations, particularly in clinical studies. Therefore, assessing MDA levels in plasma or serum provides a convenient in vivo indicator of LPO and serves as a non-invasive biomarker of OS, frequently used to study radical-mediated physiological and pathological conditions [43,68]. Because MDA and other aldehydes can modify both DNA bases and functional proteins, they may contribute to mutagenic, genotoxic, and cytotoxic events [43].

Thus, MDA has a dual relevance in oncology: it is a measurable indicator of LPO and a biologically active aldehyde potentially involved in molecular damage.

## 4. MDA in Tumors

### 4.1. Breast Cancer

Breast cancer is one of the most prevalent malignancies in women and remains among the leading causes of cancer-related mortality worldwide [69]. Several studies have investigated the relationship between breast cancer, OS, LPO, and MDA-related molecular damage, using circulating, tissue, and cytological samples. Overall, most available evidence suggests that breast cancer is associated with increased LPO and altered antioxidant defenses, although the clinical significance of MDA remains heterogeneous and method-dependent [70].

Specifically, a study aimed to assess OS in patients with breast cancer by measuring plasma levels of LPO markers, including MDA, reported significantly elevated MDA levels compared with healthy individuals [71]. Similar increases in circulating MDA were previously reported in breast cancer patients, including studies using serum or plasma samples, some of which also showed alterations in trace elements or antioxidant status. In particular, serum MDA measured by high-performance liquid chromatography was significantly increased in breast cancer patients and was positively associated with copper levels, suggesting a possible contribution of metal-related redox imbalance to lipid oxidative damage [72,73].

Other studies have also evaluated whether MDA levels vary according to disease stage. Increased LPO and impaired antioxidant enzyme activity were reported across different stages of breast cancer, with higher MDA levels in more advanced disease in selected cohorts [74]. Other data showed increased serum MDA and nitrite levels in breast cancer patients compared with controls, with a significant rise from stage III to stage IV disease, suggesting that systemic oxidative and nitrosative stress may become more pronounced in advanced-stage breast cancer [75].

The impact of OS and LPO–induced DNA damage in breast cancer has also been investigated by measuring tissue levels of the DNA adduct malondialdehyde deoxyguanosine (M1dG). MDA-derived DNA adducts provide a more direct mechanistic link between LPO and breast carcinogenesis than circulating MDA alone. Putative MDA-DNA adducts have been detected in human breast tissues, supporting the hypothesis that lipid peroxidation products may accumulate in the mammary microenvironment and generate potentially genotoxic lesions [76]. In one study, breast tissue samples were obtained by fine-needle aspiration from 35 women with either breast cancer or benign breast lesions. The results showed that M1dG adduct levels were significantly higher in malignant breast tissue than in benign tissue, with mean values more than fivefold higher in cancer samples. Moreover, elevated M1dG levels were significantly associated with more aggressive tumor features, including higher histological grade and larger tumor size. These findings suggest that OS–related DNA damage may contribute to breast cancer development and progression [77].

Additionally, another study assessed the balance between OS and antioxidant defenses in women with breast cancer by analyzing specific biochemical markers in their blood. Serum levels of total antioxidant capacity (TAC), lipid hydroperoxide, and MDA were measured. The results indicated that breast cancer patients experience a notable shift in redox status, with increased LPO markers and decreased overall antioxidant capacity compared with healthy controls. Moreover, the severity of this oxidant-antioxidant imbalance worsened with disease progression, with advanced-stage patients showing greater oxidative tissue damage and lower antioxidant defenses than those in early stages. This evidence suggests that monitoring systemic LPO products, including MDA, alongside total antioxidant status, may offer valuable biochemical insights into breast cancer progression [78].

However, available findings are not fully consistent. In a comparative study evaluating LPO and antioxidant status in blood and tissue samples from patients with malignant breast tumors and benign breast disease, serum and tissue MDA levels were lower in malignant tumors than in benign lesions [79]. This discordant result is important because it indicates that MDA levels may vary according to sample type, local antioxidant responses, tumor biology, and the comparator group used. In addition, although serum MDA levels were found to be higher in patients with invasive breast cancer than in those with benign breast disease, no significant correlation was observed with major pathohistological prognostic factors, including tumor size, histological grade, lymph node involvement, estrogen receptor status, menopausal status, or age [80]. These data argue against considering serum MDA as a reliable independent prognostic biomarker in breast cancer.

MDA has also been investigated in relation to treatment- and surgery-associated OS. After three cycles of doxorubicin/cyclophosphamide chemotherapy, serum MDA levels increased significantly, whereas total antioxidant status decreased, suggesting that MDA may capture chemotherapy-related oxidative injury. In the perioperative setting, dynamic changes in oxidant/antioxidant markers have also been observed, with MDA increasing shortly after surgery and decreasing during the postoperative period. Interestingly, higher MDA levels were reported in patients with lymph node-positive disease than in lymph node-negative patients, suggesting a possible association between LPO and more advanced tumor biology, although this finding requires further validation [81,82].

Overall, these findings highlight that MDA and MDA-related DNA adducts may serve as useful biomarkers of oxidative damage in breast cancer. However, current evidence does not support MDA as a validated diagnostic or prognostic biomarker when used alone. MDA should rather be regarded as a biologically relevant indicator of LPO and oxidative molecular damage, potentially useful as an adjunctive marker within broader redox biomarker panels. Its interpretation remains limited by methodological heterogeneity, differences between serum, plasma, tissue, and fine-needle aspiration samples, variable comparator groups, and potential confounders such as age, menopausal status, diet, inflammation, metabolic factors, and treatment exposure.

### 4.2. Lung Cancer

Despite changes in global cancer incidence patterns over recent decades, lung cancer remains the leading cause of cancer-related mortality worldwide. Established risk factors include tobacco use, air pollution, and exposure to environmental tobacco smoke. Carcinogenic and mutagenic compounds found in tobacco smoke and air pollutants can promote the formation of aromatic DNA adducts, which are implicated in lung carcinogenesis. In recent years, increasing attention has also been given to the contribution of OS and LPO of cellular membranes to the development of several malignancies, including lung cancer [83].

One study reported a trend toward improved survival in lung cancer patients with lower levels of MDA–DNA adducts, although this association did not reach statistical significance. MDA–DNA adducts have been proposed as potential systemic markers of OS, possibly reflecting biological processes related to tumor progression [84]. However, any interpretation regarding potential intervention strategies remains speculative, given the small size of the study population. Moreover, survival in lung cancer is influenced by multiple clinical factors, including comorbidities, disease stage, treatment modality, and surgical resection parameters [84,85].

Another study evaluated changes in OS markers during treatment in patients with lung cancer and assessed whether these changes were associated with treatment response and survival. The study included untreated patients with non-small-cell lung cancer receiving cisplatin and etoposide, as well as a healthy control group. Blood samples were collected at baseline and after the third and sixth chemotherapy cycles to measure OS-related parameters, including LPO products, nitric oxide, GSH levels, and superoxide dismutase activity. The results showed that disease progression was associated with increased OS and reduced antioxidant defenses. Conversely, patients who responded to chemotherapy showed lower levels of LPO products and nitric oxide, together with higher GSH levels and preserved antioxidant enzyme activity after treatment. Responders also had a significantly longer mean survival than non-responders. These findings suggest that, in patients responding to chemotherapy, systemic OS may decrease while antioxidant defenses are preserved [86].

Overall, it can be hypothesized that LPO products, particularly MDA and MDA-derived DNA adducts, may have clinical relevance as biomarkers in lung cancer. Elevated baseline levels may reflect cumulative carcinogen exposure and oxidative DNA damage during carcinogenesis, whereas treatment-related changes may provide insight into therapeutic response. Lower LPO levels appear to be associated with better chemotherapy response and longer survival, supporting the potential prognostic value of MDA-related biomarkers. Nevertheless, larger, well-characterized prospective studies are required to validate their clinical utility.

### 4.3. Head and Neck Tumors

Head and neck cancer (HNC) comprises a heterogeneous group of malignant tumors arising in the upper aerodigestive tract, including the salivary glands, oral cavity, nasopharynx, oropharynx, hypopharynx, larynx, and paranasal sinuses. Depending on tumor site, stage, and histological subtype, HNC can be treated with radiation, chemotherapy, surgery, or a combination of these approaches [87].

One commonly used chemotherapeutic agent is cisplatin, a platinum-based compound with systemic and relatively non-selective cytotoxic activity, which may therefore cause significant adverse effects. Cisplatin-induced generation of ROS contributes to apoptosis and cellular injury not only in malignant cells but also in normal tissues. This oxidative imbalance may damage healthy cells by reducing antioxidant defenses, such as superoxide dismutase (SOD), and increasing LPO markers, particularly MDA [88].

In patients with HNC, SOD activity may decrease, whereas MDA levels may increase because of cisplatin-induced ROS production. Astaxanthin is an exogenous antioxidant that may counteract oxidative stress by enhancing SOD activity and lowering MDA levels. Evidence suggests that astaxanthin supplementation can increase SOD activity and decrease MDA levels in patients with HNC receiving cisplatin-based therapy [89].

Another study investigated the impact of lifestyle habits, particularly tobacco and alcohol use, on oxidative tissue damage by measuring serum MDA levels in 90 individuals divided into three groups: healthy controls, patients with oral precancerous lesions, and patients with oral cancer. Serum MDA levels increased progressively across the groups, from 5.107 ηmol/mL in controls to 9.33 ηmol/mL in the precancerous group and 14.34 ηmol/mL in the oral cancer group, suggesting a strong association between mucosal transformation and systemic OS, thus suggesting that serum MDA may reflect increasing systemic oxidative damage across healthy, precancerous, and malignant oral conditions [90]. However, these cross-sectional findings do not establish its clinical utility for early detection, disease monitoring, or therapeutic guidance [90].

In conclusion, MDA may serve as an important marker for both lifestyle-related mucosal changes and systemic toxicity caused by treatment in HNC. The increase in serum MDA levels from healthy individuals to those with precancerous lesions and oral cancers may support its use as an accessible biomarker for early detection and disease staging. Additionally, the rise in OS and MDA during cisplatin chemotherapy may suggest its potential role as both an indicator of malignancy and a contributor to treatment-related cellular damage. Monitoring systemic MDA levels may therefore aid in diagnosing and predicting outcomes for upper aerodigestive tract tumors, while also providing a biochemical baseline to assess the effectiveness of antioxidants such as astaxanthin in reducing treatment-related toxicity.

### 4.4. Colorectal Cancer

Colorectal cancer (CRC) is a malignancy of the intestinal tract and is considered the second leading cause of cancer-related mortality worldwide. The disease is heterogeneous, arising from a complex and not yet fully understood sequence of molecular events. OS seems to play a role in carcinogenesis [91].

The involvement of OS in the development and progression of human CRC has been reported in various studies. Nevertheless, limited information is available regarding the biochemical changes in tissues and blood and how these relate to the clinical stages of the disease [92,93].

Several studies have analyzed serum MDA levels across different stages of CRC and examined the correlation between this biomarker and disease stage. These studies also evaluated the relationship between MDA levels and histopathological tumor size, as determined by preoperative, intraoperative, and histological findings. Additionally, the association between serum MDA levels and histopathological indicators of disease extent was assessed by comparing serum MDA levels with histopathological findings obtained after surgical tumor resection [94,95,96].

Specifically, a Croatian study investigated the relationship between OS, assessed through antioxidant enzyme activity, and the LPO marker MDA in CRC using various predictive models [94]. Previous research has shown that CRC patients typically exhibit lower levels of antioxidative enzymes and higher MDA levels [97]. This finding served as the basis for the study hypothesis, which aimed to determine whether similar patterns are present in the Croatian patient population. According to study results, MDA determination could be a candidate marker for further investigation as an indicator of OS intensity or LPO-related damage in CRC. An increase in MDA levels may result from cellular damage, reactions with free amino groups of proteins and nucleic acids, as well as from mutagenicity targeted at the guanine site of the DNA sequence, while also being a consequence of rapid metabolism of cancer cells in situ, contributing to MDA release into the bloodstream. Notably, two predictor variables—cancer size and MDA level—were found to be significantly correlated. The findings suggest that for each 1 cm increase in tumor size, the likelihood of progressing to a higher stage increases by approximately 2.45 times, assuming all other factors remain constant [94]. In this cohort, tumor size and MDA levels contributed to a statistical model associated with cancer stage; however, the model was not externally validated. While tumor size was more statistically significant as an independent variable, combining both variables led to a more accurate prediction model. This underscores the value of using statistical models in cancer research. The study demonstrated that a model incorporating both tumor size and MDA levels may represent a useful tool for researchers in this field [94].

Another study showed that CRC patients carrying specific genetic variants of the Glutathione S-Transferase Mu 1 (GSTM1) gene, which encodes for a critical detoxification enzyme that neutralizes environmental carcinogens and protects cells from OS, exhibited significantly higher plasma MDA levels and elevated serum copper levels compared with healthy individuals. Furthermore, these patients exhibited significantly lower serum selenium concentrations, suggesting that specific GSTM1 gene variants may influence systemic OS and trace-element imbalances during the development of CRC [98].

Ultimately, these findings suggest clear disturbances in enzymatic and non-enzymatic antioxidant systems, as well as enhanced oxidation of proteins and lipids in the serum/plasma of CRC patients. Moreover, the authors proposed that catalase and MDA may be potential non-invasive biomarkers for differentiating tumor invasion depth or indicating the occurrence of lymph node metastasis.

In summary, MDA may represent a non-invasive indicator of systemic oxidative imbalance in CRC. However, its associations with tumor size and stage-related parameters are exploratory and do not currently establish a role in staging or outcome prediction. Larger prospective studies and external validation are required.

### 4.5. Cervical Cancer

Cervical cancer is the fourth most common cancer among women worldwide, following breast, lung, and colorectal cancers. Its development is a multistep process largely driven by persistent infection with oncogenic high-risk human papillomavirus (HPV) types, which promote epithelial transformation and may lead to precancerous lesions collectively referred to as cervical intraepithelial neoplasia (CIN) [69,99,100].

In this context, OS may act as an additional cofactor in HPV-related cervical carcinogenesis by promoting LPO, antioxidant depletion, oxidative DNA damage, and a pro-inflammatory microenvironment [101].

Several studies have reported increased LPO in women with cervical cancer. Early case–control studies showed higher circulating MDA or LPO levels in cervical cancer patients than in healthy controls, together with impaired antioxidant defenses, supporting the presence of systemic oxidative imbalance in this malignancy [102]. Similar findings were reported in cervical squamous cell carcinoma, where increased plasma and erythrocyte MDA levels were accompanied by higher nitric oxide levels, suggesting the coexistence of oxidative and nitrosative stress [103]. Stage-related studies also reported increased MDA, nitric oxide, and copper levels, along with reduced antioxidant defenses, including erythrocyte superoxide dismutase activity, vitamin C, and zinc levels, suggesting that oxidative imbalance may become more pronounced during disease progression [104].

Evidence from premalignant cervical lesions further suggests that LPO may occur before invasive cancer develops. Increased Thiobarbituric Acid Reactive Substances (TBARS)/MDA levels have been reported in women with low-grade squamous intraepithelial lesions, high-grade squamous intraepithelial lesions, and cervical cancer, indicating that oxidative lipid damage may already be detectable during the premalignant stages of cervical carcinogenesis [105]. Consistently, studies on CIN showed altered antioxidant vitamin status and increased plasma MDA levels, supporting the hypothesis that oxidative imbalance accompanies early HPV-related epithelial transformation [106]. In addition, studies comparing CIN and invasive cervical carcinoma reported increased oxidative damage and impaired antioxidant defenses, further supporting a role for LPO in the transition from premalignant lesions to carcinoma [107].

A comparative cross-sectional study evaluated the relationship between serum MDA concentrations, cervical cancer, and high-risk HPV infection. The study reported significantly higher mean serum MDA levels in women with cervical cancer and in those with high-risk HPV infection compared with women without high-risk HPV. However, no significant difference in serum MDA levels was observed between women with cervical cancer and those with high-risk HPV infection alone. These findings suggest that increased systemic LPO may already be present in women with high-risk HPV infection and may persist during malignant transformation [108]. Therefore, in this setting, serum MDA appears more informative as an indicator of redox imbalance in HPV-related cervical disease than as a discriminator between high-risk HPV infection and established cervical cancer.

More recent studies have further supported the association between cervical cancer and LPO. In a cross-sectional study comparing women with cervical cancer and healthy controls, plasma MDA levels were significantly higher in cervical cancer patients, whereas total antioxidant capacity was reduced and the OS index was increased [109]. Other studies reported increased LPO, assessed as TBARS expressed as MDA, together with altered antioxidant parameters and increased 8-hydroxy-2′-deoxyguanosine levels, reinforcing the link between LPO and oxidative DNA damage in cervical cancer [110].

MDA has also been evaluated in the treatment setting. In patients undergoing chemoradiation, serum MDA has been analyzed together with total antioxidant capacity and other OS markers. These studies suggest that chemoradiation may modify the oxidant-antioxidant profile in cervical cancer patients and that oxidative damage markers, including MDA, protein carbonyls, and 8-hydroxy-2′-deoxyguanosine, may change during concurrent chemoradiotherapy. Complete responders showed dynamic treatment-related changes followed by partial normalization at follow-up, whereas this pattern was not clearly observed in non-responders, suggesting a possible relationship between redox modulation and treatment response. However, MDA should not currently be considered an independent predictive marker of chemoradiation response [111,112].

Overall, these findings support an association between high-risk HPV infection, cervical carcinogenesis, and systemic OS. Elevated MDA levels in high-risk HPV infection, CIN, and cervical cancer suggest that LPO may occur early and persist during disease progression. Nevertheless, current evidence does not support MDA as a validated stand-alone diagnostic or prognostic biomarker in cervical cancer. Its interpretation remains limited by methodological heterogeneity, the frequent use of TBARS-based assays, differences between serum, plasma, erythrocyte, and tissue-related measurements, and potential confounding factors such as HPV status, inflammation, smoking, nutritional status, disease stage, and treatment exposure. Further longitudinal studies are needed to clarify whether MDA may have clinical value as part of broader redox biomarker panels in HPV-related cervical disease.

### 4.6. Cutaneous Tumors

As a stable end-product of LPO, MDA is widely used as an indirect biomarker of OS and oxidative tissue damage. Its relevance in skin biology is supported by the high cutaneous exposure to ultraviolet radiation (UVR), chronic inflammation, mitochondrial ROS production, and pigment-related redox reactions [113].

In melanoma, several studies suggest that OS is increased both within tumor tissue and at the systemic level. In an immunohistochemical study evaluating malignant melanoma, non-melanoma skin cancer (NMSC), benign nevi, adjacent non-tumoral skin, and young control skin, Sander et al. reported increased protein-bound MDA in melanoma tissue, supporting the presence of enhanced LPO in the melanoma microenvironment [113].

Notably, MDA immunoreactivity was not restricted to malignant melanocytes but was also detected in surrounding keratinocytes, suggesting that oxidative lipid damage may extend beyond the tumor cell compartment and involve adjacent epidermal structures. This observation is consistent with the concept that melanoma develops and progresses within a redox-active cutaneous microenvironment, although it does not prove that MDA directly drives melanoma progression [113].

Circulating MDA has also been investigated in patients with melanoma. Some studies have reported increased systemic MDA levels and altered antioxidant status compared with healthy controls, with more pronounced oxidative alterations in advanced disease [114,115]. In one longitudinal study, oxidative stress parameters partially improved after surgical tumor removal, whereas plasma MDA levels increased following DTIC-based chemotherapy, suggesting that both tumor burden and treatment-related oxidative injury may influence systemic MDA concentrations [114,116]. Other clinical studies confirmed higher serum or plasma MDA levels in melanoma patients than in healthy subjects, with the highest values observed in advanced-stage disease, particularly stage IV melanoma [117,118]. These findings suggest an association between systemic LPO and melanoma progression, although available cohorts are relatively small and MDA has not been validated as an independent diagnostic or prognostic biomarker.

A melanoma-specific aspect that deserves consideration is the intrinsic redox vulnerability of melanocytes. Unlike keratinocyte-derived tumors, melanoma arises from cells in which melanogenesis itself may contribute to ROS generation. Melanin can act as both a UV-absorbing antioxidant pigment and, under specific conditions, a pro-oxidant system, particularly in the presence of melanin intermediates or pheomelanin-rich phenotypes [119]. This melanocyte-specific redox background may provide a plausible context for increased MDA levels, reflecting the convergence of UV-driven LPO, pigment-related oxidative reactions, mitochondrial metabolism, inflammation, metabolic rewiring, and therapy-induced OS. However, MDA remains a non-specific marker and may be influenced by age, smoking, diet, metabolic disease, systemic inflammation, and analytical methodology, especially when quantified using thiobarbituric acid reactive substances assays [120,121].

In NMSC, the relationship between MDA and carcinogenesis should be interpreted within the broader framework of UV-induced field cancerization. Chronic UVR exposure generates ROS, depletes cutaneous antioxidant defenses, promotes LPO, and contributes to a permissive microenvironment for keratinocyte transformation [25]. Within this context, MDA is relevant because it reflects oxidative degradation of PUFAs and because MDA-derived adducts may modify proteins and nucleic acids, thereby linking photodamage to persistent molecular dysfunction.

The strongest evidence among keratinocyte cancers concerns cutaneous squamous cell carcinoma (cSCC). In the study by Sander et al., non-melanoma tumors showed a markedly disturbed antioxidant balance, with reduced expression of antioxidant enzymes and increased protein-bound MDA, particularly in cSCC rather than in basal cell carcinoma (BCC) [113]. This distinction is biologically meaningful because cSCC usually develops through a multistep pathway involving chronically sun-damaged skin, actinic keratosis, cSCC in situ/Bowen disease, and invasive cSCC [122,123]. Consistently, Yoshifuku et al. showed that MDA expression was increased in actinic keratosis, Bowen disease, and cSCC compared with healthy controls, with the highest expression observed in SCC, suggesting that lipid oxidation may be particularly relevant in later stages of squamous carcinogenesis [124]. Thus, the cSCC-associated MDA signal is best interpreted as part of a chronic oxidative field rather than as a tumor-specific molecular alteration.

Additional support comes from experimental and translational data on MDA-derived epitopes in UV-exposed skin. Williams et al. showed that solar-simulated UVR induces free MDA and MDA-derived protein epitopes in human keratinocytes and human skin, indicating that LPO products can be generated directly after phototoxic stress in epidermal cells [125]. In the same study, MDA- and dihydropyridine (DHP)-lysine-derived epitopes were detected in NMSC specimens, and cSCC tissue showed a marked increase in both MDA- and DHP-lysine epitopes compared with adjacent non-tumoral tissue. These findings suggest that LPO in cSCC may leave persistent biochemical footprints in tumor tissue [126].

The stronger MDA-related signal observed in cSCC compared with BCC may reflect the more inflammatory and biologically aggressive nature of cSCC, which commonly arises in chronically photodamaged skin and is associated with actinic keratosis, stromal inflammation, immunosuppression, and a higher potential for invasion and metastasis [125,126]. In this context, MDA may reflect the combined effects of UVR, inflammatory oxidants, mitochondrial dysfunction, and reduced antioxidant enzyme activity, rather than acting as a direct driver of cSCC initiation or progression.

Systemic studies also suggest that patients with NMSC may display measurable oxidative imbalance, although these findings are less specific for MDA. Increased serum MDA has been reported in broad skin cancer cohorts, while studies assessing cSCC, BCC, actinic keratosis, and controls found altered redox markers, including TBARS, protein carbonyls, total antioxidant capacity, GSH, and catalase activity [127,128]. Since TBARS are commonly used as an LPO readout but are not fully specific for MDA, these data support systemic oxidative imbalance in NMSC while emphasizing the need for more specific analytical methods.

Evidence for BCC is more nuanced. BCC is strongly linked to UVR, but its biology is largely driven by aberrant Hedgehog signaling and is characterized by slow growth, local invasiveness, and very low metastatic potential. Compared with cSCC, BCC may therefore show a less intense or less consistent MDA signature, particularly at the systemic level. Local tissue studies indicate that LPO may still occur within BCC lesions, as paired analyses have shown significantly higher MDA concentrations in BCC tissue than in adjacent non-affected skin. However, serum MDA appears less informative and does not consistently correlate with tissue levels, suggesting that MDA in BCC may better reflect local oxidative imbalance than systemic disease burden [129].

Regarding Merkel cell carcinoma (MCC), direct evidence on MDA is currently very scarce. MCC comprises biologically distinct virus-positive and virus-negative subsets; virus-positive tumors are associated with Merkel cell polyomavirus, whereas virus-negative tumors generally show a high ultraviolet-associated mutational burden [130]. Because UVR can induce oxidative damage, LPO may theoretically be relevant in the redox biology of virus-negative MCC, but this remains speculative in the absence of dedicated studies measuring MDA in MCC tissue, serum, or plasma [131]. A small Taiwanese series investigated OS in MCC using 8-hydroxy-2′-deoxyguanosine (8-OHdG) rather than MDA and suggested that oxidative DNA damage may be detectable in selected MCC specimens, particularly in the context of Merkel cell polyomavirus infection. Thus, MDA should not currently be described as an established biomarker in MCC; rather, its potential role remains an unresolved research question [132].

**Figure 2:**
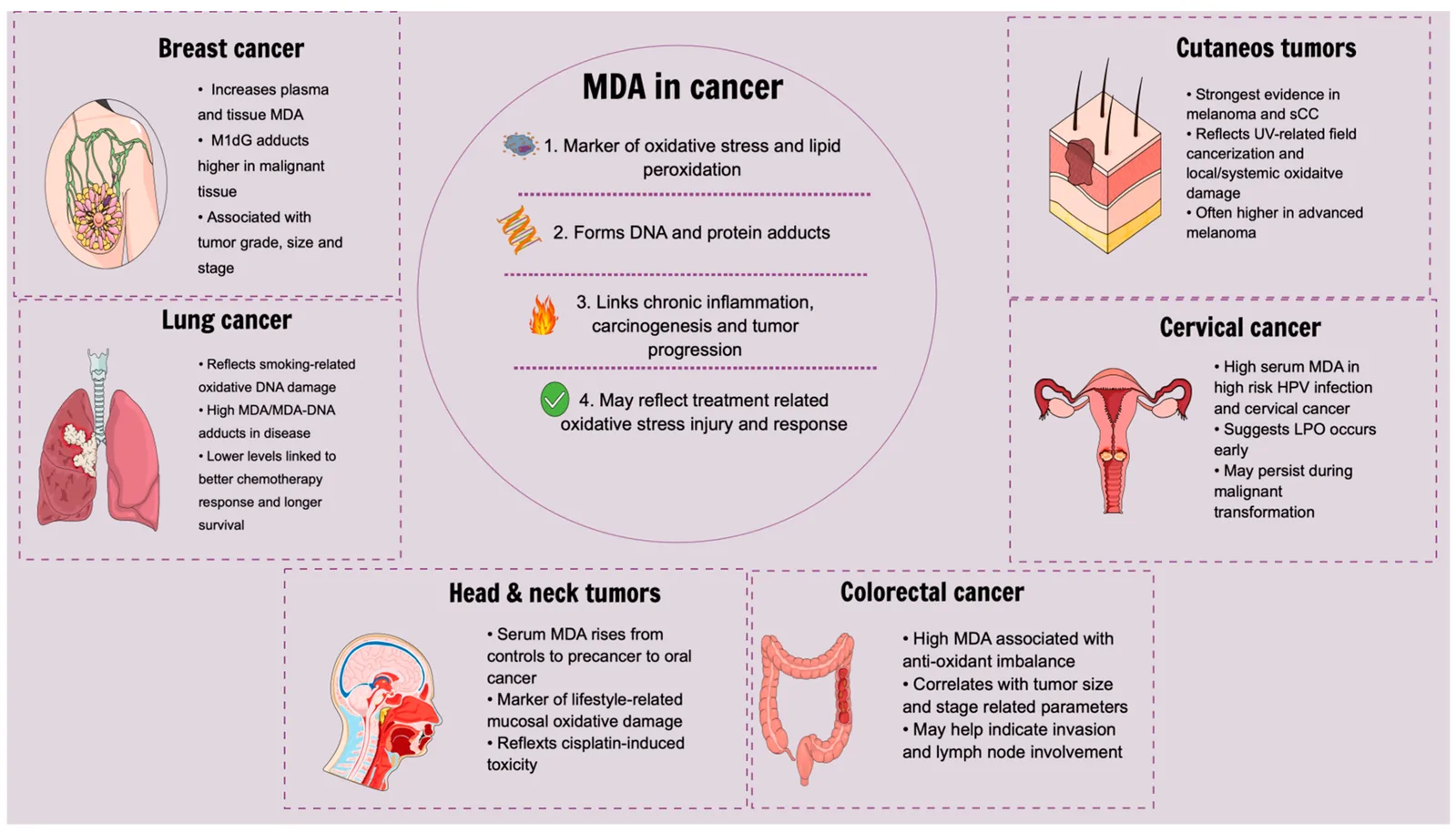
Role of malondialdehyde in cancer.

Overall, MDA represents a biologically relevant marker of LPO in cutaneous malignancies, particularly in melanoma and cSCC. Its tissue and circulating levels may reflect oxidative damage, tumor-associated redox imbalance, UV-related field cancerization, and, in some studies, disease stage. However, because MDA is non-specific and methodologically variable, it should currently be regarded as an oxidative stress marker rather than a validated diagnostic, prognostic, or therapeutic biomarker in skin cancer (Figure 2).

## 5. Diagnostic Applications and Analytical Considerations of MDA in Cancer

The interpretation of MDA measurements is strongly influenced by analytical and pre-analytical factors. Although thiobarbituric acid-reactive substances (TBARS) assays are simple and widely used, they lack specificity because thiobarbituric acid reacts with several aldehydes and other interfering compounds in addition to MDA, while the acidic and high-temperature conditions required by the assay may promote artefactual MDA formation [42]. Chromatographic methods provide greater analytical specificity; however, differences in hydrolysis and derivatization, calibration, extraction procedures, and the measurement of free or total MDA still limit comparability across studies. Measured concentrations may also be influenced by the biological matrix, anticoagulant use, processing time, storage temperature and duration, freeze–thaw cycles, and sample handling. Serum and plasma results should therefore not be pooled or directly compared without matrix- and method-specific validation [133,134,135].

MDA has been evaluated in serum, plasma, erythrocytes, saliva, tumor tissue, adjacent non-tumoral tissue, and cytological samples. Serum and plasma are the most frequently investigated matrices because they are readily accessible and suitable for repeated measurements; however, circulating MDA may reflect systemic inflammation, comorbidities, lifestyle factors, treatment exposure, or oxidative processes occurring outside the tumor [136]. Tissue measurements may potentially more closely reflect local tumor-associated lipid peroxidation but require invasive sampling and are influenced by intratumoral heterogeneity, tissue processing, and the selection of comparator tissue. Erythrocyte MDA is commonly used as an index of erythrocyte membrane lipid peroxidation and is not directly equivalent to serum or plasma MDA. Salivary MDA has been investigated particularly in oral squamous cell carcinoma. In one small case–control study, receiver operating characteristic analysis suggested better discriminatory performance for salivary than for circulating MDA, although the findings were derived from the same cohort and were not independently validated [137]. A systematic review and meta-analysis reported increased MDA levels in plasma, serum, and saliva from patients with oral squamous cell carcinoma, whereas the limited subgroup of tissue studies reported lower MDA levels than in control tissues, emphasizing the influence of the biological matrix [138].

MDA concentrations can also be influenced by cancer-independent factors, including age, sex, smoking, diet, obesity, diabetes, metabolic disorders, systemic inflammation, concomitant medication use, antioxidant supplementation, renal or hepatic dysfunction, and previous or ongoing anticancer treatment. These variables should be prospectively recorded and addressed through matching, stratification, eligibility criteria, or multivariable analyses. In longitudinal studies, sampling should also be standardized in relation to surgery, chemotherapy, radiotherapy, other systemic treatments, and acute inflammatory events. Without adequate control of these factors, differences in MDA levels cannot be confidently attributed to cancer presence, progression, or treatment response [139].

Although several case–control studies have reported differences in circulating, salivary, or tissue MDA concentrations between patients with cancer and control groups, most have assessed group-level differences rather than formal diagnostic accuracy. Receiver operating characteristic (ROC) analyses, predefined cut-off values, sensitivity, and specificity have been reported only in selected studies. In oral squamous cell carcinoma, one study suggested greater discriminatory performance for salivary than for circulating MDA, whereas another reported improved performance when MDA was combined with salivary 8-hydroxy-2′-deoxyguanosine and antioxidant vitamins rather than used alone [137,140]. In breast cancer, MDA showed some discriminatory ability but performed less well than 8-hydroxy-2′-deoxyguanosine and protein carbonyls [141]. In one colorectal cancer cohort, serum MDA yielded an area under the ROC curve of 0.82, with a sensitivity of 85.1% and a specificity of 73.1%; however, MDA concentrations were lower in patients than in controls, and the proposed threshold was not externally validated [142]. Therefore, statistically significant differences or favorable ROC results obtained in individual case–control cohorts should not be considered equivalent to established clinical diagnostic performance.

Before MDA can be considered for clinical application, analytical and pre-analytical procedures must be standardized, and matrix-, assay-, and clinical-context-specific reference intervals and thresholds should be prospectively evaluated and independently validated. MDA should also be compared directly with established cancer biomarkers and assessed in multivariable models to determine whether it provides incremental diagnostic or prognostic information beyond clinical presentation, tumor stage, histology, molecular subtype, inflammatory status, and treatment exposure. Current evidence therefore supports MDA primarily as an indicator of lipid oxidative damage rather than as a validated stand-alone diagnostic biomarker. Future studies should determine whether MDA measured using analytically specific methods can provide clinically relevant information when incorporated into multimarker panels containing tumor-specific, inflammatory, and other redox-related parameters.

## 6. Conclusions

MDA, one of the main end-products of LPO, represents a biologically relevant marker of OS and oxidative tissue damage in the oncological field.

The evidence reviewed in this article suggests that increased MDA levels are frequently observed across several malignancies, including breast, lung, head and neck, colorectal, cervical, and cutaneous ones. Overall, MDA levels have been associated with tumor presence, disease stage, oxidative DNA damage, and treatment-related toxicity, although results remain heterogeneous across cancer types.

Taken together, these findings support the potential role of MDA as an accessible, non-invasive biomarker of oxidative damage in cancer. However, current evidence does not yet support its use as a stand-alone diagnostic, prognostic, or therapeutic biomarker. MDA is not tumor-specific and may be influenced by several confounding factors, as well as by treatment exposure and analytical methodology. In addition, many available studies are cross-sectional, include relatively small cohorts, and use heterogeneous biological samples and measurement techniques.

Future research should therefore focus on large, prospective, and well-characterized clinical studies designed to evaluate MDA in relation to tumor burden, stage, treatment response, recurrence, and survival. Standardization of analytical methods is essential, particularly because commonly used assays, such as TBARS, may lack specificity for MDA. MDA should also be assessed using more specific methods and as part of broader redox biomarker panels integrated with clinical and molecular data. These approaches may clarify its potential value in patient stratification and in monitoring tumor- and treatment-related oxidative injury.

## Figures and Tables

**Table 1:** Major ROS-regulated pathways in cancer and their relevance to LPO and MDA.

Pathway/Axis	ROS-Mediated Regulation	Main Effects in Cancer	Relevance to LPO/MDA
EGFR–RAS–RAF–MEK–ERK	Oxidation of EGFR and inhibition of protein tyrosine phosphatases	Proliferation and survival	Indirect
PI3K–AKT–mTOR	Reversible oxidative inactivation of PTEN	Growth, survival, and metabolic adaptation	Indirect
KEAP1–NRF2	Oxidative modification of KEAP1 and NRF2 stabilization	Antioxidant adaptation and treatment resistance	May limit LPO and MDA formation
HIF-1α–VEGF	ROS-dependent HIF-1α stabilization under hypoxia	Metabolic adaptation and angiogenesis	Indirect and context-dependent
ATM–p53	Oxidative activation of ATM and DNA-damage signaling	Cell-cycle arrest, senescence, or apoptosis	Indirect
System Xc^−^–GSH–GPX4	Detoxification of phospholipid hydroperoxides	Protection from ferroptosis	Direct control of LPO and MDA precursors

Abbreviations: ATM, ataxia-telangiectasia mutated; EGFR, epidermal growth factor receptor; GPX4, glutathione peroxidase 4; GSH, glutathione; HIF-1α, hypoxia-inducible factor 1-alpha; KEAP1, Kelch-like ECH-associated protein 1; LPO, lipid peroxidation; MDA, malondialdehyde; NRF2, nuclear factor erythroid 2-related factor 2; PTEN, phosphatase and tensin homolog; VEGF, vascular endothelial growth factor.

## Data Availability

No new data were created or analyzed in this study. Data sharing is not applicable to this article.
